# Comparative Evaluation of Triladyl^®^ and Andromed^®^ Extenders Combined with Pre-Freezing Cooling Methods for Ram Semen Cryopreservation

**DOI:** 10.3390/vetsci13090991

**Published:** 2026-09-19

**Authors:** Abdallah M. Shahat, Montana Williams, Taeja Brisby, Joshua Woodard, Niki C. Whitley, Mahipal Singh, Brou Kouakou, Adel R. Moawad

**Affiliations:** 1 Reproductive Physiology Laboratory, Animal Science Program, College of Agriculture, Family Sciences, and Technology, Fort Valley State University, Fort Valley, GA 31088, USA; abdallah.mohamed@fvsu.edu (A.M.S.);; 2 Department of Theriogenology, Faculty of Veterinary Medicine, Cairo University, Giza 12211, Egypt

**Keywords:** ram, sperm, motility, semen extender, cryopreservation, cooling, packaging

## Abstract

Cryopreservation can adversely affect the quality of ram spermatozoa, reducing their suitability for artificial insemination. Therefore, optimizing cryopreservation protocols, including extender composition and cooling procedures, is important for improving post-thaw sperm quality. Our aim was to assess the effects of two commercially available extenders (Triladyl^®^ and Andromed^®^) and two cooling methods before freezing (cooling in 15 mL conical tube versus cooling in straws) on the quality of cryopreserved ram semen. Semen samples collected from five mature Katahdin rams once/week for four weeks were pooled and diluted in either Andromed^®^ or Triladyl^®^ to a final concentration of 800 × 10^6^ spermatozoa/mL. Samples were allocated into four treatment groups in a 2 × 2 factorial design: Triladyl^®^ package-then-cool (T1), Triladyl^®^ cool-then-package (T2), Andromed^®^ package-then-cool (A1), and Andromed^®^ cool-then-package (A2). In the cool-then-package method, semen was cooled in 15 mL conical tubes before being loaded into 0.25 mL French straws, whereas in the package-then-cool method, semen was first loaded into the straws and then cooled. All groups were kept at 4 °C for 3 h before freezing. Samples were subsequently frozen in a programmable freezer (MiniDigitcool, IMV^®^), stored in liquid nitrogen, and thawed later for analysis. Following thawing, sperm motility (using CASA), viability and abnormalities (using trypan-blue stain), membrane integrity (by hypos-osmotic swelling (HOS) test), acrosome status (by Hoechst 33342/FITC-PNA), mitochondrial activity (using Hoechst 33342/Rhodamin 123), and lipid peroxidation (using BODIPY 581/591 C11) were evaluated. Total motility, acrosome status, mitochondrial activity, and lipid peroxidation showed extender effects (*p* < 0.05). However, progressive motility, and membrane integrity showed cooling effects (*p* < 0.05). Moreover, among the four treatment groups, the percentages of motile sperm, live sperm, sperm with intact acrosomes, and sperm with active mitochondria were the highest (*p* < 0.05) in the Triladyl^®^ cool-then-package (T2; 31.1 ± 2.1, 26.7 ± 2.3, 66.3 ± 1.9, 76.2 ± 1.1, and 78.0 ± 1.2, respectively) group. Meanwhile, the percentages of abnormal and lipid peroxidation-positive sperm were the lowest (*p* < 0.05) in the same group (18.0 ± 0.7 and 12.3 ± 1.0, respectively). In conclusion, cryopreservation of ram semen using the Triladyl^®^ extender in combination with the cool-then-package method resulted in superior post-thaw sperm quality than other extender and cooling procedures.

## Simple Summary

Cryopreservation is widely used in sheep breeding; however, the freezing and thawing process can impair sperm function and reduce fertilizing capacity. In this study, we evaluated the effects of two commercial semen extenders (Triladyl^®^ and Andromed^®^) and two pre-freezing cooling strategies on post-thaw ram sperm quality. Semen diluted with Triladyl^®^ and cooled in 15 mL tubes before packaging into straws exhibited superior post-thaw motility, viability, acrosome integrity, and mitochondrial activity compared with semen diluted in Andromed^®^ and cooled directly in straws. These results indicate that both extender selection and cooling methodology significantly influence the quality of cryopreserved ram semen. Optimizing these procedures may enhance post-thaw sperm function and improve the effectiveness of artificial insemination programs in sheep.

## 1. Introduction

Cryopreservation of ram semen is a key component of modern sheep reproductive technology, allowing long-term genetic conservation and efficient artificial insemination (AI) operations [1]. However, ram spermatozoa are particularly sensitive to cooling- and/or cryopreservation-induced damage such as cold shock, osmotic stress, and oxidative injuries resulting in lower sperm quality and function [2,3,4]. This can be attributed to the elevated levels of polyunsaturated fatty acids (PUFAs), and the low intramembrane cholesterol-to-phospholipid ratio as well as the low antioxidant defenses in sperm plasma membranes [2].

These deleterious effects can be mitigated by employing semen extenders during cryopreservation. Semen extenders provide biochemical protection by supplying energy substrates, buffering capacity, cryoprotectants, and membrane-stabilizing components [3,4]. Commercial extenders such as Triladyl^®^ (egg yolk-based) and Andromed^®^ (soybean lecithin-based) are widely used in sperm cryopreservation because of their standardized composition and proven cryoprotective properties [5,6,7,8,9]. Egg yolk has been a key component of semen extenders due to its ability to safeguard sperm membranes during cryopreservation. This process is attributed to interactions with the plasma membrane lipid bilayer that stabilize membrane structure and reduce phase-transition damage, particularly in small animal species [10]. Accordingly, extenders containing Tris and egg yolk, together with milk-based diluents, have formed the basis of numerous effective semen cryopreservation strategies [10,11,12]. However, the use of egg yolk presents several disadvantages, including variability in composition rendering various challenges in standardizing extender formulations, increased risk of microbial contamination, and the potential presence of harmful metabolites or endotoxins that may detrimentally impact sperm viability and motility [10,11]. To overcome these limitations, plant-derived alternatives, particularly soybean lecithin, have been developed. As a plant-derived source of phosphatidylcholine and essential fatty acids, including stearic, oleic, and palmitic acids, soybean lecithin contributes to maintaining sperm membrane stability during freezing and thawing. As a result, Andromed^®^ offers the advantage of eliminating animal-derived ingredients while maintaining effective sperm protection [10,11,13,14]. Nevertheless, even with optimized extender formulations, post-thaw sperm recovery remains suboptimal in many cases, suggesting that additional procedural factors during semen handling may critically influence cryosurvival [15,16,17,18,19].

Among the procedural parameters affecting cryopreservation outcomes, the pre-freezing cooling method is a major determinant of sperm survival in various livestock species, including rams, bulls, and stallions. The pre-freezing cooling strategy directly influences membrane stability, osmotic balance, and the likelihood of intracellular ice crystal formation during cryopreservation [19,20,21,22]. For example, precision-controlled cooling technologies have been shown to effectively alleviate the cold shock commonly associated with rapid pre-freeze cooling of stallion semen, thereby improving post-thaw sperm quality and viability. This protective effect is largely attributed to enhanced stabilization of the plasma membrane lipid bilayer, which reduces phase-transition damage and preserves membrane integrity during cooling and freezing [23,24]. Suboptimal cooling rates can induce cold shock, causing membrane destabilization, protein conformational changes, ion transport disruption, increased ROS production, and reduced mitochondrial membrane potential, ultimately impairing sperm function [25,26]. In rams, controlled cooling before programmable freezing improves cryosurvival compared with uncontrolled cooling by promoting a gradual and uniform temperature decline [27,28]. The pre-freezing handling strategy, specifically cooling before packaging versus packaging before cooling, may further influence cooling kinetics and the physicochemical environment surrounding spermatozoa [24]. These differences can affect membrane stability and cellular responses during cryopreservation, thereby influencing post-thaw sperm quality and survival.

Despite these theoretical implications, experimental evidence comparing pre-freezing cooling strategies in ram semen cryopreservation remains limited, and their interaction with extender type is poorly understood. Addressing this knowledge gap is important for developing cryopreservation protocols that are both biologically effective and practically efficient. We hypothesized that extender type, pre-freezing cooling method, and their interaction influence post-thaw sperm quality. Therefore, this study evaluated the effects of two commercial semen extenders (Triladyl^®^ and Andromed^®^) combined with two pre-freezing cooling methods (cooling in 15 mL conical tubes or cooling in straws) on post-thaw ram sperm quality.

## 2. Materials and Methods

### 2.1. Rams, Semen Collection, and Initial Evaluation

All procedures involving animals were reviewed and approved by the Fort Valley State University Agricultural and Laboratory Animal Care and Use Committee (ALACUC; Protocol No. SU-R-01-2024). Five sexually mature Katahdin rams aged 2 to 3 years were housed under standard management conditions at Fort Valley State University, Fort Valley, Georgia, USA. The study was performed during the natural breeding season between October and November 2025. Rams were fed a commercial lamb ration (Godfrey’s Feed SGLF Show & Grow Lamb Feed; Godfrey’s Warehouse Inc., Madison, GA, USA) containing a minimum of 16% crude protein, 2.4% crude fat, and a maximum of 13% crude fiber. The diet was supplemented with lasalocid (20 g/ton) for coccidiosis control. Water was provided ad libitum. Semen was collected from each ram once weekly by electroejaculation for four consecutive weeks, as previously described [29]. Immediately after collection, semen quality was assessed using a computer-assisted sperm analysis (CASA) system with AndroVision^®^ software version 1.2.2 (Minitube, Verona, WI, USA). Total motility (TM), progressive motility (PM), and sperm concentration were determined after dilution (1:100) in Triladyl^®^ extender (Minitube, Verona, WI, USA). Briefly, a 15 µL aliquot of diluted semen was loaded onto a microscope slide, covered with a coverslip, and evaluated under a 20× phase-contrast objective lens. Five randomly selected fields were analyzed per sample. Sperm concentration was expressed as the number of spermatozoa per milliliter of semen. Only ejaculates meeting the minimum quality thresholds of ≥80% TM, ≥70% PM, and ≥2.0 × 10^9^ sperm/mL were included in the study. To minimize individual-animal variation, qualified ejaculates from the five rams collected during each sampling session were pooled. Each pooled ejaculate was divided into four equal aliquots corresponding to the experimental treatments and processed according to the designated cryopreservation protocol. The experiment followed a 2 × 2 factorial design consisting of two semen extenders (AndroMed^®^ and Triladyl^®^) and two cryopreservation approaches (package-then-cool and cool-then-package), resulting in four treatment groups. The pooled ejaculate served as the experimental unit, whereas the four weekly semen collections represented independent biological replicates for statistical analysis.

### 2.2. Extender Preparation, Semen Dilution, Cooling, and Cryopreservation

Two commercial extenders, AndroMed^®^ and Triladyl^®^ (Minitube, Verona, WI, USA), were used for semen dilution and prepared according to the manufacturer’s instructions. AndroMed^®^ was prepared by gentle mixing of 200 mL of the extender with 800 mL of Milli-Q water prewarmed to 35 °C. The Triladyl^®^ egg yolk extender was prepared using fresh egg yolk obtained from commercially sourced, white-shelled chicken eggs. The egg yolk was carefully separated from the albumen using filter paper to remove residual egg white. After puncturing the vitelline membrane, the yolk content was transferred into a sterile Falcon tube while avoiding contamination with membrane fragments or albumen residues. The prepared yolk was subsequently warmed and used for extender preparation. Triladyl^®^ was prepared by mixing 250 mL of Triladyl^®^ in 750 mL of Milli-Q water, followed by the addition of 5% (*v*/*v*) freshly prepared egg yolk at 30 °C. The mixture was gently mixed and sterile filtered before use. Both extenders were maintained at 37 °C until semen processing.

Each pooled semen sample was divided into four equal aliquots. Two aliquots were diluted with Triladyl^®^ extender and the remaining two with AndroMed^®^ extender to achieve a final sperm concentration of 800 × 10^6^ spermatozoa/mL. Following dilution and prior to cooling, sperm TM and PM were assessed. No differences were detected among treatment groups at this stage, confirming comparable semen quality before cryopreservation.

For the package-then-cool groups, semen aliquots were loaded into 0.25 mL French straws (IMV, L’Aigle, France), ultrasonically sealed (MRS1 Dual v2, IMV), placed horizontally on racks, and cooled from 30 °C to 4 °C in a refrigerator over 3 h at a rate of approximately 0.17 °C/min.

For the cool-then-package groups, 5 mL semen aliquots were transferred to 15 mL conical tubes and cooled from 30 °C to 4 °C in a water bath placed in a refrigerator over 3 h at approximately 0.14 °C/min. After cooling, semen was packaged into 0.25 mL straws, ultrasonically sealed, placed horizontally on racks, and equilibrated at 4 °C for an additional 15 min before freezing.

Straws from all treatment groups were cryopreserved using a programmable freezer (MiniDigitcool, IMV, L’Aigle, France) according to the following cooling profile: −5 °C/min from 4 to −10 °C, −40 °C/min from −10 to −65 °C, −30 °C/min from −65 to −100 °C, and −20 °C/min from −100 to −140 °C [30]. Upon completion of the freezing program, straws were immediately plunged into liquid nitrogen (−196 °C) and stored for one week. For post-thaw evaluation, straws were thawed in a 37 °C water bath for 30 s. Thawed semen was transferred to prewarmed microcentrifuge tubes and assessed for sperm motility, kinematic parameters, viability, morphological abnormalities, plasma membrane integrity, acrosome integrity, mitochondrial activity, and lipid peroxidation.

#### Experimental Groups

The experimental treatments were divided into four groups based on extender type and pre-freezing cooling procedure: T1, semen was diluted with Triladyl^®^ followed by packaging and then cooling (package-then-cool); T2, semen diluted with Triladyl^®^ was subjected to cooling prior to packaging (cool-then-package); A1, semen diluted with Andromed^®^ was subjected to packaging then cooling (package-then-cool); and A2, semen diluted with Andromed^®^ was cooled prior to packaging (cool-then-package).

### 2.3. Assessment of Sperm Motility and Kinematic Parameters

The percentage of total and progressive sperm motility, and kinematic parameters, including curvilinear velocity (VCL; µm/s), straight-line velocity (VSL; µm/s), average path velocity (VAP; µm/s), curved line distance (DCL; µm), straight-line distance (DSL, µm), amplitude of lateral head displacement (ALH, µm), linearity (LIN), and straightness (STR), were assessed as previously described [31]. Briefly, a 15 µL aliquot of frozen–thawed semen was placed onto a microscope slide, covered with a coverslip, and analyzed using the CASA system equipped with AndroVision^®^ software version 1.2.2 (Minitube, Verona, WI, USA). Sperm motion characteristics were evaluated under a 20× phase-contrast objective lens. For each sample, a minimum of five randomly selected microscopic fields were recorded and analyzed. All CASA settings were standardized and optimized for ram sperm and kept constant throughout the study [31].

### 2.4. Assessment of Sperm Viability and Morphological Abnormalities

The percentages of live and abnormal sperm were assessed with trypan blue staining (MP Biomedicals, Solon, OH, USA) as previously described [32,33]. Briefly, 20 µL of thawed semen was mixed with an equal volume of 0.4% trypan blue and smeared onto a clean glass slide. After air drying, slides were examined under a phase-contrast microscope (Leica DM6 B microscope system, Leica Microsystems, Wetzlar, Germany) at 40× magnification. For each sample, a total of 100 spermatozoa were evaluated across multiple randomly selected microscopic fields. Spermatozoa exhibiting complete or intense staining were classified as non-viable (dead), whereas unstained and partially stained (half-stained) spermatozoa were classified as viable (live). Viability was expressed as the percentage of live spermatozoa relative to the total number of cells counted. Sperm morphological abnormalities were evaluated simultaneously and included head defects (pyriform head, tapered head, and knobbed acrosome), midpiece defects (distal midpiece reflex, bowed midpiece, and mitochondrial sheath defects), and tail defects (coiled tail, bent tail, and detached head). The percentage of abnormal spermatozoa was calculated based on the evaluation of 100 sperm cells per sample.

### 2.5. Assessment of Sperm Plasma Membrane Integrity

The integrity of the sperm plasma membrane was determined through a hypo-osmotic swelling (HOS) test as previously described [34]. Briefly, 50 µL of thawed semen was mixed with 1 mL of hypo-osmotic solution (125 mOsm/L) consisting of 1.351 g fructose and 0.753 g sodium citrate dihydrate dissolved in 100 mL of Milli-Q water. The mixture was incubated at 37 °C for 45 min to induce osmotic swelling in spermatozoa with functionally intact plasma membranes. Following incubation, a 15 µL aliquot of the suspension was placed on a microscope slide, covered with a coverslip, and examined under a phase-contrast microscope at 40× magnification. A total of 100 spermatozoa were evaluated per sample across multiple microscopic fields. Sperm exhibiting characteristic tail swelling or curling (HOS-positive) were classified as having intact plasma membranes. Plasma membrane integrity was expressed as the percentage of HOS-positive spermatozoa relative to the total number of cells counted.

### 2.6. Assessment of Sperm Acrosome Integrity

Acrosome integrity was examined by the Hoechst 33342/FITC-PNA staining kit (6.6%; Minitube, Verona, WI, USA) as previously described [31]. Briefly, 25 µL of thawed semen was mixed with 14 µL of the staining solution in a microcentrifuge tube and incubated in the dark at 37 °C for 20 min. Following incubation, a 10 µL aliquot of the stained suspension was placed on a clean glass slide and covered with a coverslip. Samples were examined using a fluorescence microscope (Leica DM6 B; Leica Microsystems, Wetzlar, Germany) equipped with a GFP filter set (excitation: 450–490 nm; emission: 500–550 nm) and a 40× objective lens. For each sample, 100 spermatozoa were evaluated across multiple randomly selected microscopic fields. Spermatozoa exhibiting bright green fluorescence localized to the acrosomal region were classified as having an intact acrosome, whereas sperm lacking a distinct fluorescent acrosomal signal were considered acrosome-reacted or damaged [31]. Acrosome integrity was expressed as the percentage of spermatozoa with intact acrosomes relative to the total number of cells examined.

### 2.7. Assessment of Mitochondrial Activity

Mitochondrial activity was assessed using a Hoechst 33342/Rhodamine 123 staining kit (0.5%; Minitube, Verona, WI, USA) according to the manufacturer’s instructions. Briefly, 50 µL of thawed semen was mixed with 4 µL of the ready-to-use staining solution and incubated at 37 °C for 20 min in the dark. Following incubation, samples were centrifuged at 800× *g* for 4 min, and the supernatant was carefully removed. The sperm pellet was resuspended in 25 µL phosphate-buffered saline (PBS) and incubated at 37 °C for 5 min to remove excess stain. Subsequently, a 10 µL aliquot of the stained suspension was placed on a clean glass slide, covered with a coverslip, and examined using a fluorescence microscope (Leica DM6 B; Leica Microsystems, Wetzlar, Germany) equipped with a GFP filter set (excitation: 450–490 nm; emission: 500–550 nm) and a 40× objective lens. For each sample, 100 spermatozoa were evaluated across multiple randomly selected microscopic fields. Spermatozoa exhibiting bright green fluorescence in the midpiece region were classified as having active mitochondria, whereas cells lacking midpiece fluorescence were considered to have inactive mitochondria [31]. Mitochondrial activity was expressed as the percentage of spermatozoa with fluorescently labeled midpieces relative to the total number of cells examined.

### 2.8. Assessment of Lipid Peroxidation

Lipid peroxidation was evaluated using the fluorescent probe BODIPY™ 581/591 C11 (Thermo Fisher Scientific, Waltham, MA, USA) according to the manufacturer’s instructions. Briefly, 1 µL of a 200 µM stock solution was added to 100 µL of thawed semen to obtain a final probe concentration of 2 µM and incubated at 37 °C for 30 min in the dark. Following incubation, samples were centrifuged at 800× *g* for 4 min, and the supernatant was carefully discarded. The sperm pellet was then resuspended in 25 µL of PBS to remove excess probe. Subsequently, a 15 µL aliquot of the stained suspension was placed on a clean glass slide, covered with a coverslip, and examined using a fluorescence microscope (Leica DM6 B; Leica Microsystems, Wetzlar, Germany) equipped with a GFP filter set (excitation: 450–490 nm; emission: 500–550 nm) and a 40× objective lens. For each sample, 100 spermatozoa were evaluated across multiple randomly selected microscopic fields. Spermatozoa exhibiting green fluorescence in the midpiece region were classified as lipid peroxidation-positive cells, indicating oxidative damage to membrane lipids. Lipid peroxidation was expressed as the percentage of lipid peroxidation-positive spermatozoa relative to the total number of cells examined.

### 2.9. Statistical Analysis

Data were obtained from four independent experimental replicates (n = 4). Prior to statistical analysis, data were assessed for normality and homogeneity of variance. Normal distribution of residuals and equality of variances were confirmed using appropriate diagnostic procedures, including Levene’s test. A general linear model (GLM; univariate procedure) was used to evaluate the main effects of extender type (AndroMed^®^ vs. Triladyl^®^), cooling method (package-then-cool vs. cool-then-package), and their interaction (extender × cooling method). When significant effects were detected, means were compared among treatment groups using one-way analysis of variance (ANOVA) followed by Tukey’s multiple comparison test. Results are presented as mean ± standard error of the mean (SEM). Differences were considered statistically significant at *p* < 0.05. All statistical analyses were performed using IBM SPSS Statistics version 27.0 (IBM Corp., Armonk, NY, USA).

## 3. Results

### 3.1. Impact of Extender Type and Pre-Freezing Cooling Method on Post-Thaw Sperm Motility and Kinematic Parameters

**Figure 1:**
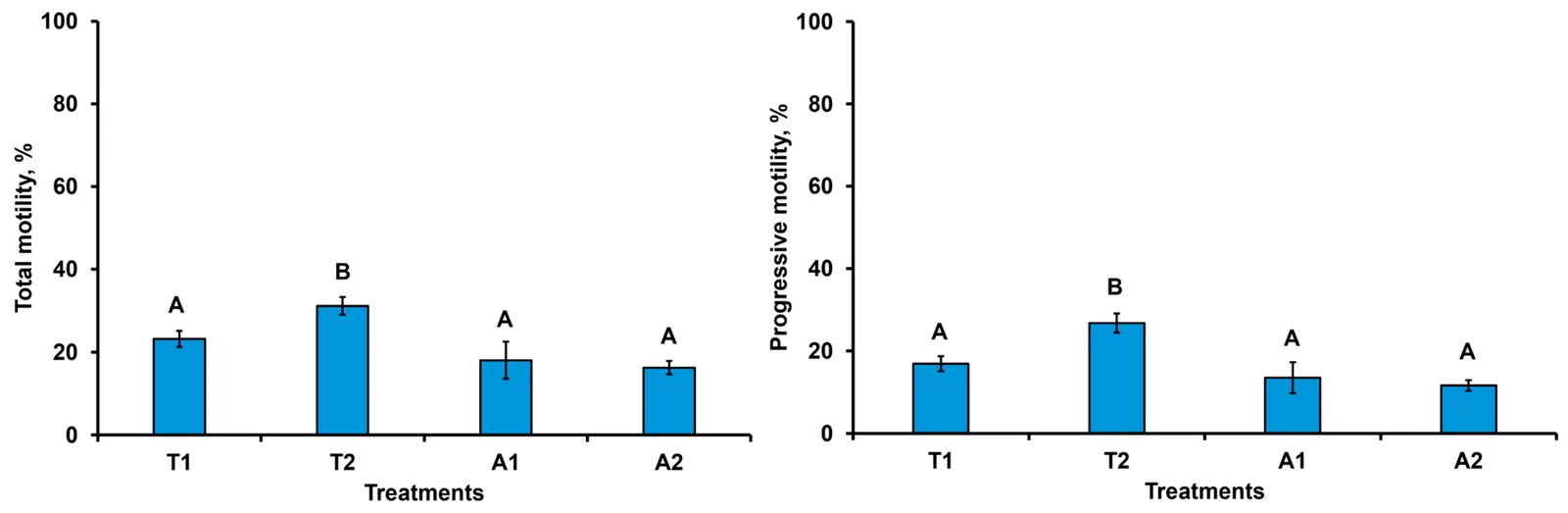
Effects of semen extender type and pre-freezing cooling method on total and progressive motility of frozen–thawed ram spermatozoa. A–B denote significant differences (*p* < 0.05). Total motility had an extender effect (*p* = 0.003), while progressive motility had a cooling effect (*p* = 0.01). T1, semen diluted with Triladyl^®^ followed by packaging and then cooling (package-then-cool); T2, Triladyl^®^-diluted semen subjected to cooling prior to packaging (cool-then-package); A1, semen diluted with Andromed^®^ followed by packaging and then cooling (package-then-cool); and A2, Andromed^®^-diluted semen cooled prior to packaging (cool-then-package).

Sperm TM and PM were greater (*p* < 0.05) in semen diluted with Triladyl^®^ extender and subjected to cooling before packaging (T2; 31.1 ± 2.1% and 26.7 ± 2.3%, respectively) than other treatment groups (Figure 1). Total motility had an extender effect (*p* = 0.003), while cooling and extender*cooling interaction effects were not significant (*p* = 0.1 and *p* = 0.7, respectively). Meanwhile, PM had a cooling effect (*p* = 0.01); on the other hand, extender and extender*cooling interaction effects were not significant (*p* = 0.9 and *p* = 0.3, respectively). No significant differences were observed (*p* > 0.05) in sperm kinematic parameters including VCL, VSL, VAP, DCL, DSL, ALH, LIN, and STR, among the different treatment groups (Table 1).

### 3.2. Effect of Extender Type and Pre-Freezing Cooling Method on Post-Thaw Sperm Viability and Total Abnormalities

**Figure 2:**
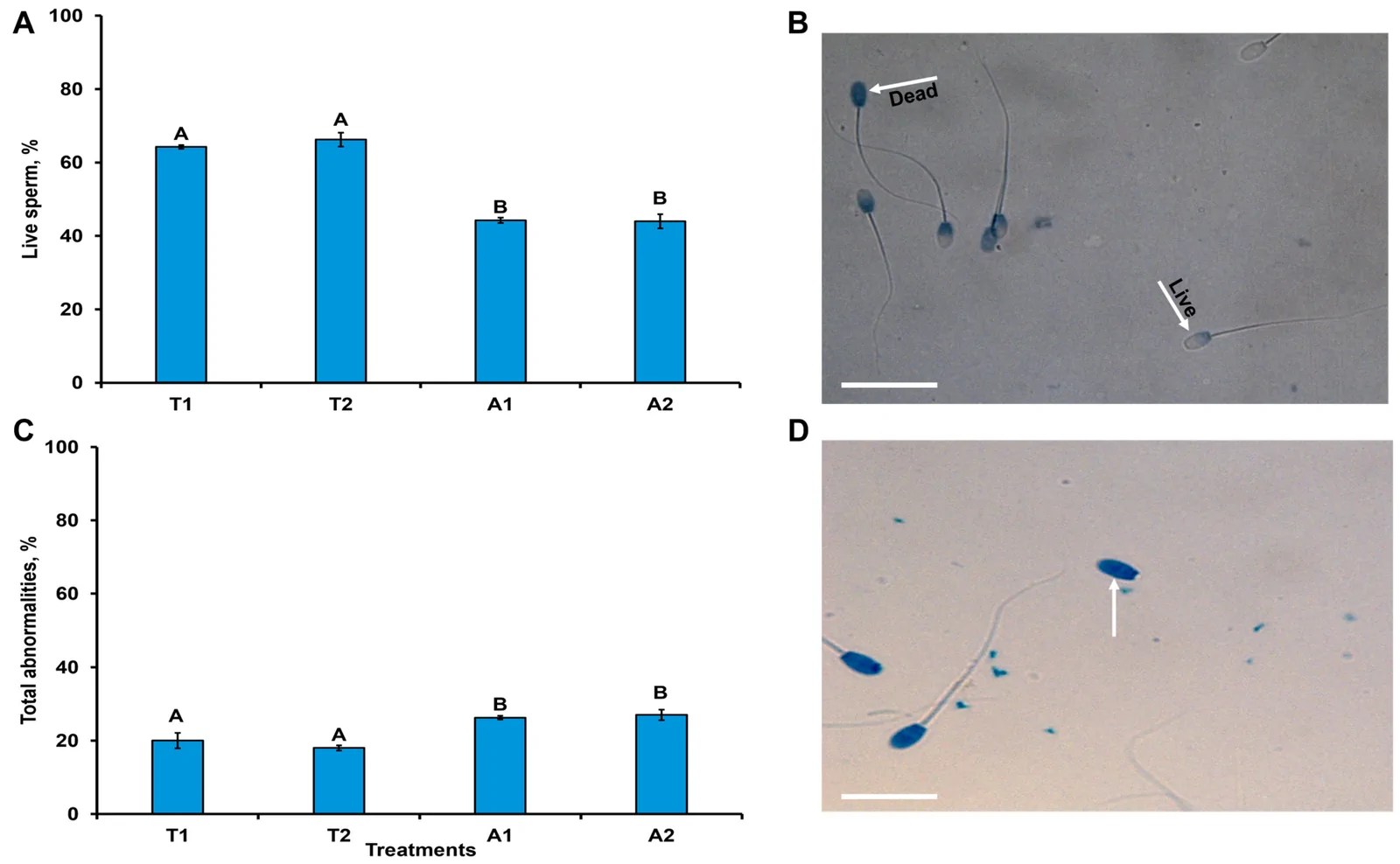
(**A**,**C**) Effects of semen extender type and pre-freezing cooling method on sperm viability and total abnormalities of frozen–thawed ram spermatozoa. A–B denote significant differences (*p <* 0.05). Sperm viability and total abnormalities had an extender effect (*p* = 0.0001). T1, semen diluted with Triladyl^®^ followed by packaging and then cooling (package-then-cool); T2, Triladyl^®^-diluted semen subjected to cooling prior to packaging (cool-then-package); A1, semen diluted with Andromed^®^ followed by packaging and then cooling (package-then-cool); and A2, Andromed^®^-diluted semen cooled prior to packaging (cool-then-package). (**B**) Representative image of post-thawed ram spermatozoa stained with trypan blue, showing live and dead spermatozoa (white arrows), scale bar = 25 µm. (**D**) Representative image of post-thawed ram spermatozoa stained with trypan blue illustrating morphological abnormalities (white arrow indicates a detached head), scale bar = 25 µm.

As presented in Figure 2, the proportions of live sperm were greater (*p* < 0.05) in T1 (Triladyl^®^ package-then-cool, 64.3 ± 0.5%) and T2 (Triladyl^®^ cool-then-package, 66.3 ± 1.9%) than A1 (Andromed^®^ package-then-cool, 44.2 ± 0.8%) and A2 (Andromed^®^ cool-then-package, 44.0 ± 1.9). However, total sperm abnormalities were lower (*p* < 0.05) in T1 (20.0 ± 2.1%), and T2 (18.0 ± 0.7%) than A1 (26.3 ± 0.5%), and A2 (27.0 ± 1.4%; Figure 2). Sperm viability and total abnormalities had an extender effect (*p* = 0.0001), while cooling and extender*cooling interaction effects were non-significant (*p* = 0.2 and *p* = 0.5, respectively).

### 3.3. Effect of Extender Type and Pre-Freezing Cooling Method on Post-Thaw Sperm Membrane Integrity

**Figure 3:**
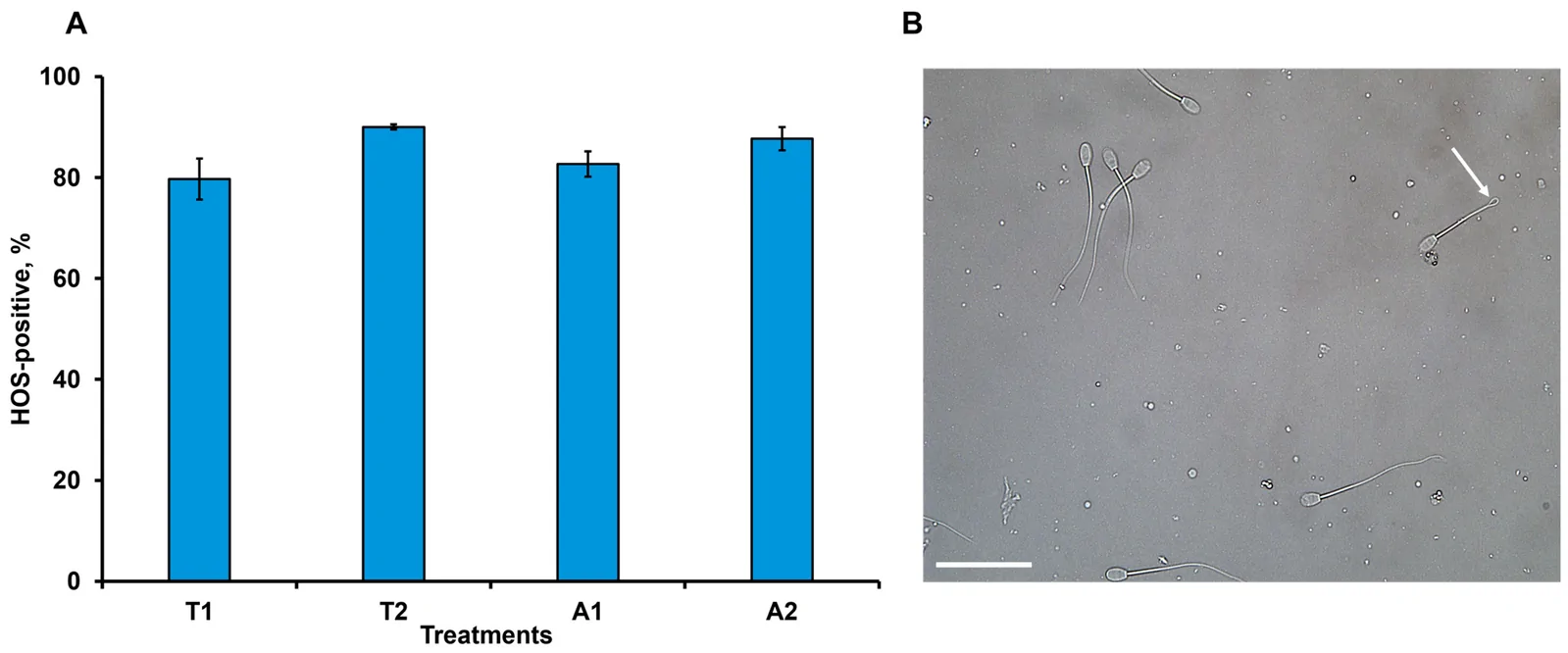
(**A**) Effects of semen extender type and pre-freezing cooling method on post-thaw ram sperm membrane integrity as evaluated by hypo-osmotic swelling (HOS) test. No significant differences were reported in sperm membrane integrity among treatment groups (*p >* 0.05). Sperm membrane integrity had a cooling effect (*p* = 0.03). T1, semen diluted with Triladyl^®^ followed by packaging and then cooling (package-then-cool); T2, Triladyl^®^-diluted semen subjected to cooling prior to packaging (cool-then-package); A1, semen diluted with Andromed^®^ followed by packaging and then cooling (package-then-cool); and A2, Andromed^®^-diluted semen cooled prior to packaging (cool-then-package). HOS: Hypo-osmotic swelling. (**B**) Image illustrating ram spermatozoa with coiled tail (HOS-positive; white arrow; intact membrane)**,** scale bar = 25 µm.

As presented in Figure 3, no significant differences (*p* > 0.05) were observed in the percentages of spermatozoa with intact membranes (HOS-positive) among the treatment groups. However, a significant effect of cooling method on sperm membrane integrity (*p* = 0.03) was observed, while extender and extender*cooling interaction effects were non-significant (*p* = 0.3 and *p* = 0.8, respectively), indicating an overall influence of cooling method despite the absence of significant pairwise differences among groups.

### 3.4. Effect of Extender Type and Pre-Freezing Cooling Method on Post-Thaw Sperm Acrosome Integrity

**Figure 4:**
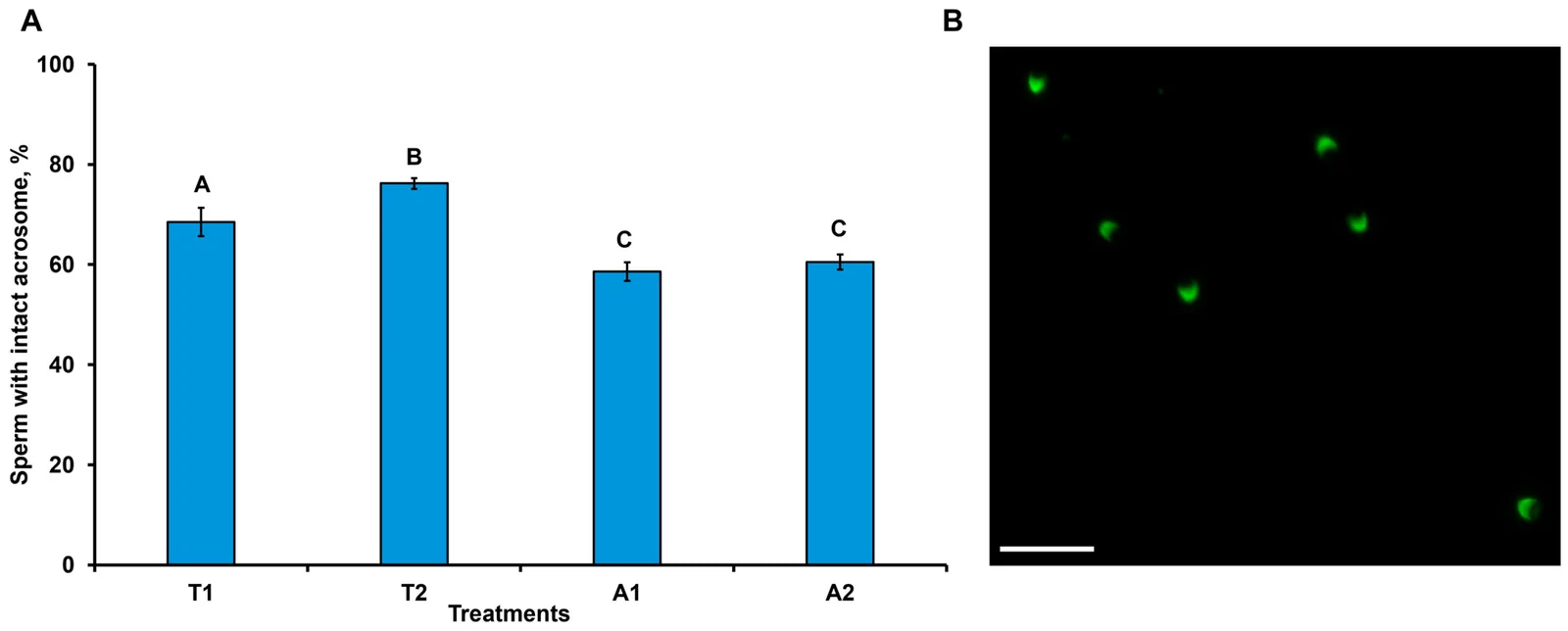
(**A**) Effects of semen extender type and pre-freezing cooling method on acrosome status of frozen–thawed ram spermatozoa. A–C denote significant differences (*p <* 0.05). Sperm with intact acrosomes had an extender effect (*p* = 0.001). T1, semen diluted with Triladyl**^®^** followed by packaging and then cooling (package-then-cool); T2, Triladyl**^®^**-diluted semen subjected to cooling prior to packaging (cool-then-package); A1, semen diluted with Andromed**^®^** followed by packaging and then cooling (package-then-cool); and A2, Andromed**^®^**-diluted semen cooled prior to packaging (cool-then-package). (**B**) Image illustrating ram spermatozoa with an intact acrosome, characterized by intense and uniform green fluorescence over the acrosomal cap following FITC-PNA staining, scale bar = 25 µm.

As shown in Figure 4, the proportions of sperm with intact acrosomes were greater (*p* < 0.05) in T2 (Triladyl^®^ extender and subjected to cooling before packaging; 76.2 ± 2.4%) than other groups (68.5 ± 9.0%, 58.6 ± 4.2%, and 60.5 ± 3.0% for T2, A1, and A2, respectively). Acrosome status had an extender effect (*p* = 0.001), while cooling and extender*cooling interaction effects were non-significant (*p* = 0.9 and *p* = 0.2, respectively).

### 3.5. Effect of Extender Type and Pre-Freezing Cooling Method on Post-Thaw Sperm Mitochondrial Activity

**Figure 5:**
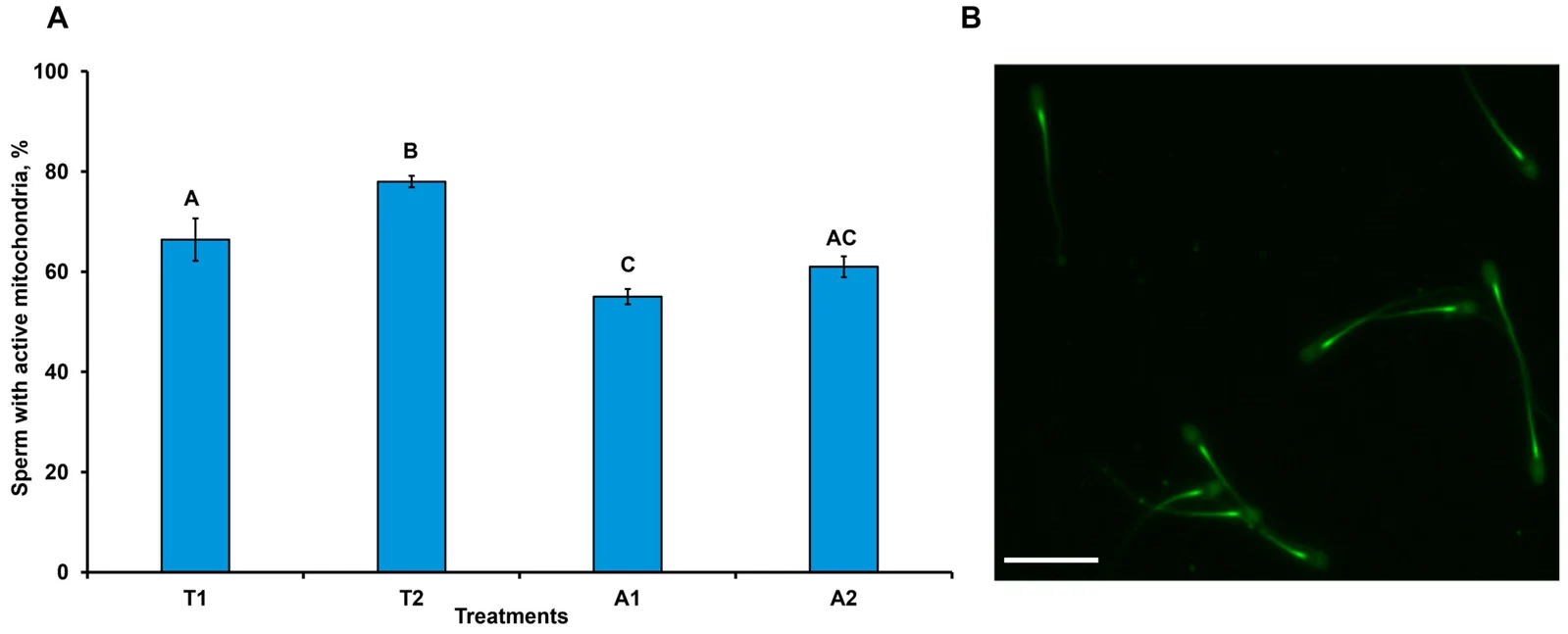
(**A**) Effects of semen extender type and pre-freezing cooling method on mitochondrial activity of frozen–thawed ram spermatozoa. A–C denote significant differences (*p <* 0.05). Sperm with active mitochondria had an extender effect (*p* = 0.001). T1, semen diluted with Triladyl^®^ followed by packaging and then cooling (package-then-cool); T2, Triladyl^®^-diluted semen subjected to cooling prior to packaging (cool-then-package); A1, semen diluted with Andromed^®^ followed by packaging and then cooling (package-then-cool); and A2, Andromed^®^-diluted semen cooled prior to packaging (cool-then-package). (**B**) Image illustrating ram spermatozoa with active mitochondria, showing bright green fluorescence in the midpiece following Hoechst 33342/Rhodamine 123 staining, scale bar = 25 µm.

As presented in Figure 5, the proportions of spermatozoa with active mitochondria were higher (*p* < 0.05) for the T2 (Triladyl^®^ extender and subjected to cooling before packaging; 78.0 ± 2.0%) group than the other ones (66.4 ± 9.5%, 55.0 ± 2.7%, 61.0 ± 3.6% for T1, A1, and A2, respectively). Mitochondrial activity had an extender effect (*p* = 0.001), while cooling and extender*cooling interaction effects were non-significant (*p* = 0.5 and *p* = 1.0, respectively).

### 3.6. Effect of Extender Type and Pre-Freezing Cooling Method on Post-Thaw Sperm Lipid Peroxidation

**Figure 6:**
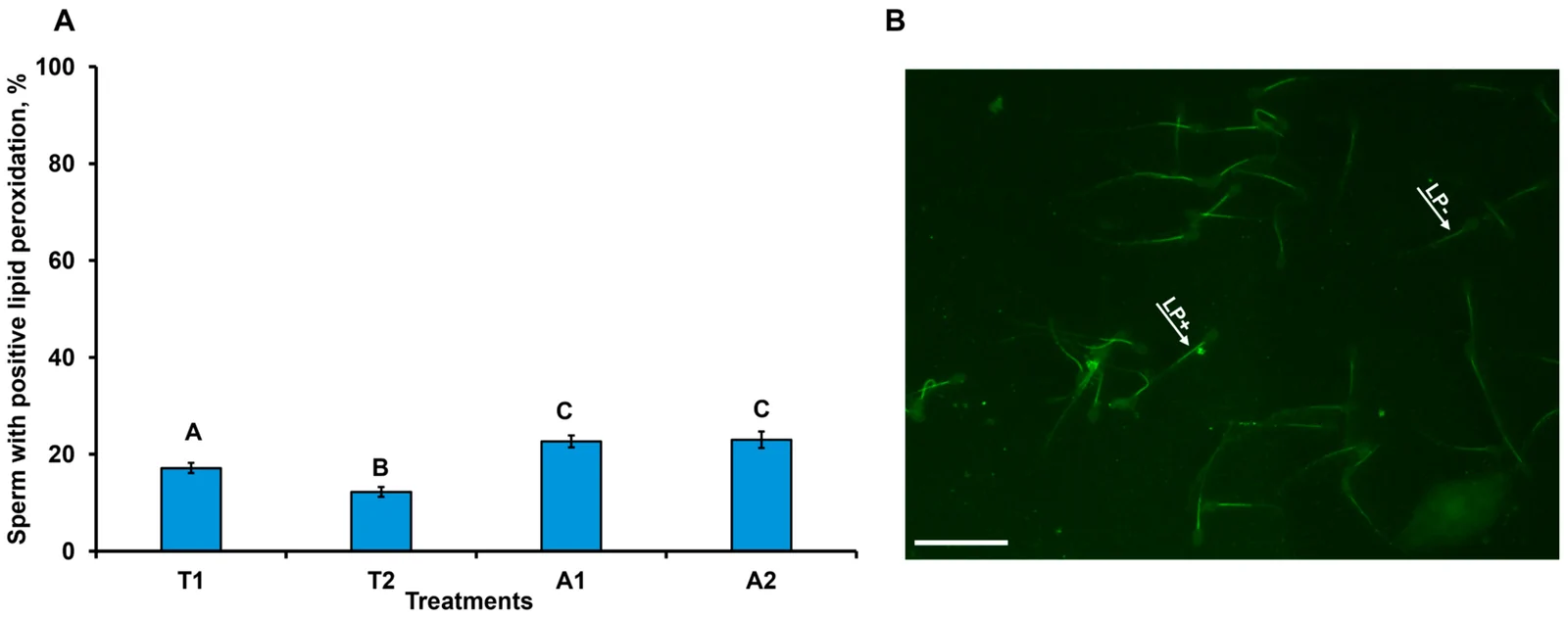
(**A**) Effects of semen extender type and pre-freezing cooling method on lipid peroxidation of frozen–thawed ram spermatozoa. A–C denote significant differences (*p <* 0.05). Sperm lipid peroxidation had an extender effect (*p* = 0.002). T1, semen diluted with Triladyl^®^ followed by packaging and then cooling (package-then-cool); T2, Triladyl^®^-diluted semen subjected to cooling prior to packaging (cool-then-package); A1, semen diluted with Andromed^®^ followed by packaging and then cooling (package-then-cool); and A2, Andromed^®^-diluted semen cooled prior to packaging (cool-then-package). (**B**) Image illustrating lipid peroxidation-positive ram spermatozoa (LP+), showing bright green fluorescence in the midpiece following BODIPY 581/591 C11 probe staining; LP- represents lipid peroxidation-negative spermatozoa that had no green fluorescence in the midpiece, scale bar = 25 µm.

As shown in Figure 6, among the four treatment groups, the lowest proportion of spermatozoa with positive lipid peroxidation was reported in the T2 group (Triladyl^®^ extender and subjected to cooling before packaging; 12.3 ± 2.1%). This value was lower (*p* < 0.05) than those seen in T1 (17.2 ± 2.7%), A1 (22.7 ± 2.1%), and A2 (23.0 ± 3.4%). Lipid peroxidation had an extender effect (*p* = 0.002), while cooling and extender*cooling interaction effects were non-significant (*p* = 0.7 and *p* = 0.06, respectively).

## 4. Discussion

Egg yolk is a non-permeable cryoprotectant widely used in tris-based extenders for bull and ram semen cryopreservation due to its ability to mitigate cold shock and preserve sperm motility, acrosome integrity, and mitochondrial activity [35]. Soybean lecithin- and liposome-based extenders represent promising alternatives to egg yolk-based formulations. In addition to extender type, pre-freezing cooling strategy, particularly the sequence of cooling and packaging, may influence cooling kinetics and the physicochemical environment surrounding spermatozoa [24]. Our findings demonstrate that both extender type and pre-freezing cooling method influence the quality of frozen–thawed ram spermatozoa. Specifically, semen diluted in Triladyl^®^ and cooled in 15 mL conical tubes before packaging in straws (cool-then-package, T2) exhibited superior post-thaw quality compared with semen diluted in AndroMed^®^ and subjected to the package-then-cool approach. These results highlight a significant interaction between extender type and cooling strategy in determining post-thaw ram sperm quality.

The superior post-thaw sperm quality observed in the T2 group, including higher total and progressive motility, viability, membrane and acrosome integrity, and mitochondrial activity, along with lower sperm abnormalities and lipid peroxidation, suggests that combining Triladyl^®^ with cooling in a conical tube before packaging in straws effectively preserves ram sperm function during cryopreservation. This benefit is likely related to reduced thermal stress and improved membrane stability [1]. In contrast, these parameters were generally lower in the AndroMed^®^ groups (A1 and A2), highlighting the importance of extender composition in sperm cryosurvival. Regardless of cooling method, the higher viability and lower sperm abnormalities observed in T1 and T2 compared with A1 and A2 further support the protective role of egg yolk against cryodamage [36,37]. Egg yolk is widely used in semen extenders because its phospholipids and low-density lipoproteins (LDL) protect the sperm plasma membrane and acrosome during cooling and freezing [35,38,39]. These components may also form lipoprotein complexes with spermatozoa, reducing cold shock and associated membrane damage [35]. When combined with glycerol, as in Triladyl^®^, this protective effect may be further enhanced [36].

Although animal-derived cryoprotectants may adversely affect semen quality due to compositional variability and the risk of microbial contamination [11], plant-based alternatives such as soybean lecithin have been developed. However, their effectiveness has been inconsistent across studies. Consistent with our findings, several reports have demonstrated the superiority of Triladyl^®^ over AndroMed^®^ in preserving ram sperm quality. For example, Hegedűšová et al. [40] found that while both extenders maintained similar sperm viability during the first 48 h of storage, Triladyl^®^ sustained sperm activity more effectively during extended preservation and produced the highest viability after 96 h. Similarly, Rekha et al. [8] reported higher sperm motility and viability in ram semen stored with Triladyl^®^ compared with other Tris-based extenders. In contrast, AndroMed^®^ has generally shown performance comparable to, but not superior to, traditional egg yolk-based extenders. For example, Fukui et al. [41] reported no significant differences in sperm function between AndroMed^®^ and Tris-based extenders.

Sperm cryosurvival is influenced by several interrelated factors, including cooling rate, equilibration period, and freezing method [4,8,16,24]. Despite optimized protocols, only about half of motile sperm typically survive the freeze–thaw process due to cryoinjury incurred during cooling and freezing [24,42,43,44]. This highlights the importance of optimizing both extender composition and pre-freezing handling procedures to improve post-thaw sperm quality. In the present study, semen diluted in Triladyl^®^ and cooled in 15 mL conical tubes before packaging (T2) exhibited the highest post-thaw quality, confirming that both extender type and cooling strategy play critical roles in ram semen cryopreservation. The superior performance of the T2 group may be explained by improved heat transfer dynamics and cooling uniformity [42]. Cooling semen in bulk before packaging allows a more homogeneous and controlled temperature decline, minimizing thermal gradients before exposure to the rapid cryogenic heat fluxes associated with straw freezing [43,44]. Consequently, the cool-then-package approach may enhance sperm survival by stabilizing membrane structures before freezing, thereby reducing sperm abnormalities and lipid peroxidation while preserving mitochondrial activity, motility, viability, and acrosome integrity, as observed in the present study.

Several limitations of the present study should be considered when interpreting the findings. First, the experiment was conducted using semen collected from a relatively small number of rams (n = 5) over four collection periods, and ejaculates were pooled before processing. Although pooling reduced individual-animal variability and ensured sufficient semen volume for the experimental procedures, it prevented the evaluation of sire-specific effects and may limit the wider applicability of the results. Second, the assessment of cryopreservation outcomes was based exclusively on in vitro sperm quality parameters. While post-thaw motility, viability, plasma membrane integrity, acrosome integrity, mitochondrial activity, and lipid peroxidation are widely accepted indicators of sperm quality, they may not fully reflect fertilizing capacity under practical breeding conditions. Therefore, additional studies incorporating in vivo fertility trials and/or in vitro embryo production systems are warranted to determine whether the improvements observed in post-thaw sperm quality translate into enhanced reproductive performance.

## 5. Conclusions

In conclusion, the use of Triladyl^®^ in combination with the cool-then-package method improved several post-thaw ram sperm quality parameters, including motility, viability, acrosome integrity, and mitochondrial activity. These results suggest that optimizing both extender type and cooling procedure may enhance the cryopreservation outcomes of ram semen.

## Figures and Tables

**Table 1:** Effects of semen extender type and pre-freezing cooling method on kinematics parameters of frozen–thawed ram spermatozoa (mean ± SEM).

Parameter	T1	T2	A1	A2
VCL (µm/s)	125.4 ± 7.5	117.3 ± 12.9	130.3 ± 5.5	135.2 ± 15.4
VSL (µm/s)	72.1 ± 4.5	63.6 ± 6.1	74.1 ± 5.2	74.6 ± 7.3
VAP (µm/s)	68.4 ± 6.9	69.3 ± 6.5	79.6 ± 4.6	80.5 ± 7.5
DCL (µm)	27.9 ± 3.2	27.6 ± 3.2	19.5 ± 6.0	30.5 ± 8.0
DSL (µm)	14.1 ± 1.2	13.5 ± 1.6	9.4 ± 2.0	14.6 ± 4.1
DAP (µm)	32.1 ± 16.7	15.1 ± 1.8	10.4 ± 2.5	16.2 ± 4.3
ALH (µm)	1.3 ± 0.1	1.2 ± 0.1	1.1 ± 0.2	1.3 ± 0.2
LIN	0.5 ± 0.02	0.6 ± 0.02	0.6 ± 0.03	0.6 ± 0.02
STR	0.9 ± 0.01	0.9 ± 0.01	0.9 ± 0.02	0.9 ± 0.01

None of the evaluated sperm kinematic parameters differed significantly among the treatment groups (*p* > 0.05). VCL (curvilinear velocity), VSL (straight line velocity), VAP (average path velocity), DCL (distance curved line), DSL (distance straight line), ALH (amplitude of lateral head displacement), LIN (linearity), STR (straightness). T1, semen diluted with Triladyl^®^ followed by packaging and then cooling (package-then-cool); T2, Triladyl^®^-diluted semen subjected to cooling prior to packaging (cool-then-package); A1, semen diluted with Andromed^®^ followed by packaging and then cooling (package-then-cool); and A2, Andromed^®^-diluted semen cooled prior to packaging (cool-then-package).

## Data Availability

The original contributions presented in this study are included in the article. Further inquiries can be directed to the corresponding author.
